# Hybrid Printed Energy Harvesting Technology for Self-Sustainable Autonomous Sensor Application

**DOI:** 10.3390/s19030728

**Published:** 2019-02-11

**Authors:** Sangkil Kim, Manos M. Tentzeris, Apostolos Georgiadis

**Affiliations:** 1 Department of Electronics Engineering, Pusan National University, Busan 46241, Korea; 2 Department of Electrical and Computer Engineering, Georgia Institute of Technology, 777 Atlantic Dr. NW, Atlanta, GA 30332, USA; etentze@ece.gatech.edu; 3 Heriot Watt University, Edinburgh Campus, Edinburgh EH14 4AS, UK; apostolos.georgiadis@ieee.org

**Keywords:** inkjet printing, hybrid printed electronics, energy harvesting, self-sustainable wireless sensor

## Abstract

In this paper, the far-field energy harvesting system for self-sustainable wireless autonomous sensor application is presented. The proposed autonomous sensor system consists of a wireless power supplier (active antenna) and far-field energy harvesting technology-enabled autonomous battery-less sensors. The wireless power supplier converts solar power to electromagnetic power in order to transfer power to multiple autonomous sensors wirelessly. The autonomous sensors have far-field energy harvesters which convert transmitted RF power to voltage regulated DC power to power-on the sensor system. The hybrid printing technology was chosen to build the autonomous sensors and the wireless power suppliers. Two popular hybrid electronics technologies (direct nano-particle printing and indirect copper thin film printing techniques) are discussed in detail.

## 1. Introduction

Self-sustainable autonomous sensor system has gained increasing popularity as the concept of the Internet-of-things (IoT) spreads in everyday life. Energy harvesting technology is one of the most important key features to realize true self-sustainable autonomous stand-alone sensor systems [1,2,3]. It is possible to operate the deployed wireless sensor system without batteries once the energy harvesting technology is enabled. It results in almost semi-permanent operating time of the wireless sensor system without any other maintenance cost. There are many types of harvestable energy in the ambient environment, such as heat, vibration and light. However, RF far-field energy harvesting technology has attracted huge attention of many researchers because RF power can be delivered wirelessly to power-hungry devices [4]. The ambient RF power density (e.g., TV signal, WiFi, or mobile, etc.) is too low to turn-on multiple microcontroller-based sensors at the same time without the assistance of batteries. Therefore, a wireless power supplier such as an active antenna is required to increase the ambient RF power density. The self-sustainable stand-alone sensors are able to harvest ambient electromagnetic power to drive analog/digital sensor systems when exposed to high RF power density. 

An additive fabrication process like inkjet printing technology has been reported in numerous research efforts since it is a flexible, low-cost, scalable, and environmentally friendly manufacturing method for next generation electronics [5]. Microwave passive components (antennas, capacitors, inductors, etc.) and nano-structures (microfluidics, MEMs, nano-materials, etc.) have been successfully manufactured by the printing technology [6,7,8]. However, it is challenging to implement fully printed high-performance ICs (integrated circuits) due to the relatively low electron mobility values and long channel length of printed transistor (relatively low printing resolution of 50 μm) [8]. To overcome the limitations (system integrity and functionality) of fully printed electronics, hybrid printing technology has been proposed that integrates commercial off-the-shelf components and printed interconnects [9,10,11]. The hybrid printing technology allows us to take full advantage of both well-matured CMOS (complementary metal–oxide–semiconductor) ICs and printed circuits.

In next section, the concept of hybrid printed electronics for self-sustainable autonomous sensor system applications is introduced in detail. A solar-to-RF power converter for wireless power transfer and an energy harvesting system for the stand-alone sensor system are presented. Popular hybrid printing technologies (nano-particle printing and indirect catalyst ink printing) are also shown in the following section. 

## 2. Hybrid Printed Electronic Technology for Autonomous Sensor Applications

**Figure 1:**
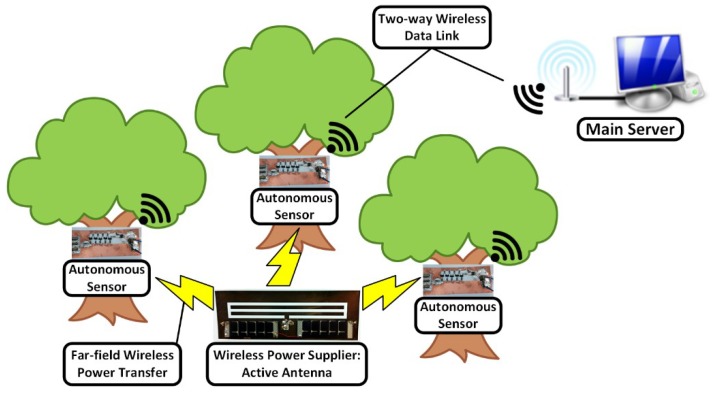
Application scenario of self-sustainable autonomous sensor system.

The concept of the proposed self-sustainable autonomous wireless sensor system is presented in Figure 1. A wireless power supplier generates RF power and increases the ambient electromagnetic power density in order to wake-up the RF energy harvesting technology-enabled sensors. In this system topology, a solar-to-RF power converting active antenna is working as the wireless power supplier. Autonomous sensors harvest electromagnetic power transmitted from the wireless power supplier to drive microcontroller-based sensor nodes. The data collected by the autonomous sensors are sent to the main server for signal processing and data analysis. The presented system does not require a battery to operate the wireless sensor system. The autonomous sensor consists of many circuit components such as the sensor, rectifier, and transmitter. The autonomous wireless sensor which consists of all the circuit components requires relatively high power (more than 10 mW) at transmitting state to drive all the circuit components. Most of the power is consumed by an RF transmitter which usually requires 1.8 V or higher bias voltage and tens of milli-amperes at 900 MHz Tx mode. It requires a high-power wireless transfer technique to drive such autonomous wireless sensor devices. Therefore, ultra-low power communication techniques such as backscattering communication or RFID (radio-frequency identification)-enabled sensor technology are popular for the autonomous sensor platform technology [12,13,14,15]. As a proof of concept, this paper presents a far-field energy harvesting system which generates wireless energy from renewable energy and delivers sufficient power to drive target sensor devices like a microcontroller because the proposed system is scalable to high or low power applications.

**Figure 2:**
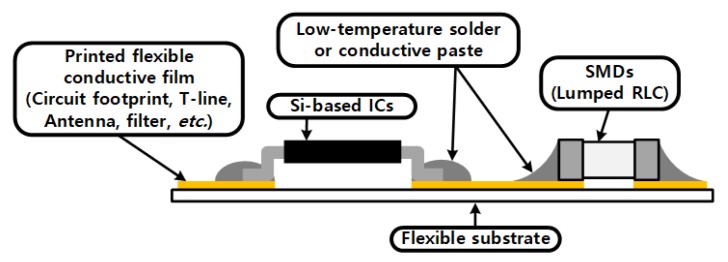
A typical topology of flexible hybrid printed electronics.

In this work, hybrid printing technology was chosen to implement the proposed autonomous wireless sensor system since hybrid printing technology takes advantage of printing technologies and well-developed surface mounting technology (SMT). It also lowers the manufacturing cost of flexible circuits (the merits of printing technology) and provides high performance of highly integrated advanced ICs (the benefits of SMT). A typical topology for hybrid printed electronics is shown in Figure 2. It consists of printed circuit layout, passive components and/bonded surface mount devices (SMDs). The fabrication process of the hybrid printed electronics consists of two steps: (1) printing a conductive layer and (2) mounting electronic devices and components (IC chip, capacitor, inductor, etc.). In this section, two major metallization techniques for the hybrid printed electronics are presented: direct silver nano-particle printing and indirect copper thin film printing (electroless electroplating) techniques.

### 2.1. Direct Silver Nano-Particle Printing: Wireless RF Power Supplier

**Figure 3:**
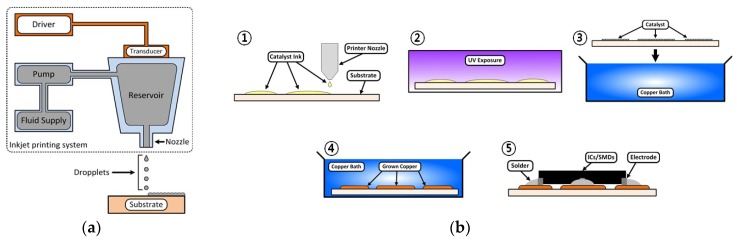
Fabrication process of printed flexible conductive film: (**a**) direct inkjet printing of silver nano-particles and (**b**) indirect printing of thin copper (Cu) film (electroless electroplating).

In this process, a piezoelectric-controlled nozzle deposits 10 pL droplets on the paper substrate ($\varepsilon_{r}=3.0\sim 3.2,\;\tan\delta =0.02\sim 0.05$, thickness = 254 μm). The fabrication process is shown in Figure 3a. The five layers of silver nano-particle ink were printed, and the printed pattern was cured in a thermal oven at 130 °C. The ink properties such as viscosity, surface tension, and particle density were modified to optimize the printing accuracy and electrical properties. This method features the advantages of relatively high conductivity and versatile substrate selection, but it is not compatible with the soldering process. Conductive silver epoxy was applied to mount SMDs and IC chips on printed silver nano-particle film.

**Figure 4:**
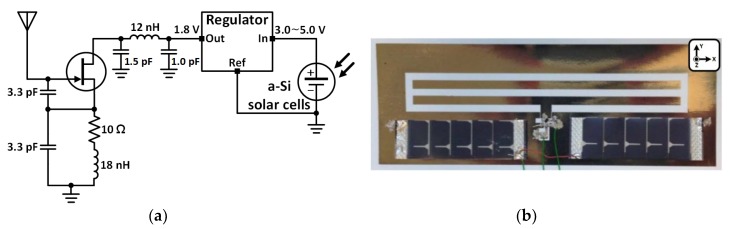
A solar-powered active antenna (solar-to-RF power converter): (**a**) System schematic and (**b**) fabricated RF active antenna.

**Figure 5:**
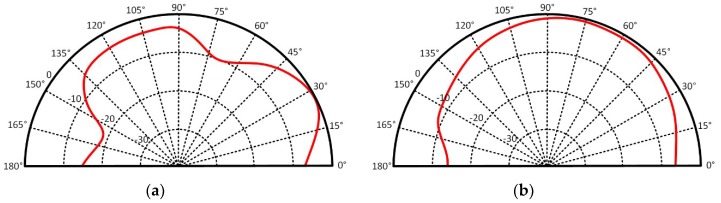
Measured radiation patterns: (**a**) E-plane (xz-plane) and (**b**) H-plane (yz-plane).

This process has been successfully used to implement complex systems, including active components. For instance, an inkjet-printed active antenna on paper for wireless power transmission and identification has been reported [9]. It verified the potential of drop-on-demand (DoD) inkjet printing technology for active paper electronics. The fabricated solar-powered active antenna operates at the UHF RFID frequency band and it consists of a slot antenna, a voltage-controlled oscillator, a voltage regulator, and solar cells. The system schematic and the fabricated active antenna is shown in Figure 4. The solar cells provide DC power to the voltage regulator, and the regulator supplies DC power to the oscillator. A printed slot antenna radiates the RF power generated by the oscillator to increase ambient RF power density (wireless RF power supplier). The oscillation frequency can be scaled up to any desired operation frequency. For the stable oscillation, a 1.8 V voltage regulator was utilized to supply the power to the oscillator. The measured phase noise of the solar-powered active antenna was about −118 dBc/Hz at 1 MHz away from the carrier frequency. The measured EIRP of the designed active antenna was about 24 dBm and the normalized radiation patterns on E-/H- plane are shown in Figure 5. 

### 2.2. Indirect Copper Film Printing: RF Energy Harvesting System for Autonomous Wireless Sensors

To realize a practical large-scale system, it is important to develop a robust fabrication process that is compatible with surface mounting technology. For this reason, an alternate technology process consisting of a combination of inkjet printing technology using a PdCl_2_ catalyst ink and electroless electroplating has been developed [10]. The catalyst-based electroless electroplating technology allows electronic components to be mounted with low temperature paste while maintaining the benefits of printing technology, such as cost efficiency and flexibility. The fabrication steps of the catalyst-based copper deposition process used in this paper are shown in Figure 3b. Palladium-based catalyst ink (PdCl_2_) was printed on a flexible low-cost polymer substrate as a seed layer. In this work, Melinex (Dupont Teijin Film, VA, USA [16]) was chosen due to its relatively high thermal durability and printability. The printed catalyst ink was exposed to UV light to improve the bond between the catalyst and the substrate. The UV exposed substrate was put in a copper bath for copper deposition. The thickness of the conductive copper layer was a function of the reaction time in the copper bath. The reported copper thickness (50 min in the copper bath) was about 4 μm and the conductivity value was about $2\times 10^{6}\sim 3\times 10^{6}\;S/m$. 

Electronic components were mounted and soldered on the deposited thin copper layer using a low temperature solder paste. It was critical to choose solder paste which was able to melt at a lower temperature than the melting point or glass transition temperature of the substrate in order to minimally damage the printed circuit footprint and the substrates. Lead-free paste consisting of Bi_58_Sn_42_ was chosen as the low temperature solder paste because it has the low melting temperature of 130 °C. For the assembly (soldering) process, it was important to control the thermal profile to form a uniform quality of solders. For instance, too low temperatures would result in inadequate flow of the paste and unstable contacts. However, too high temperatures could cause a short circuit or damaged printed conductive layers due to excessive flow of the solder paste.

**Figure 6:**
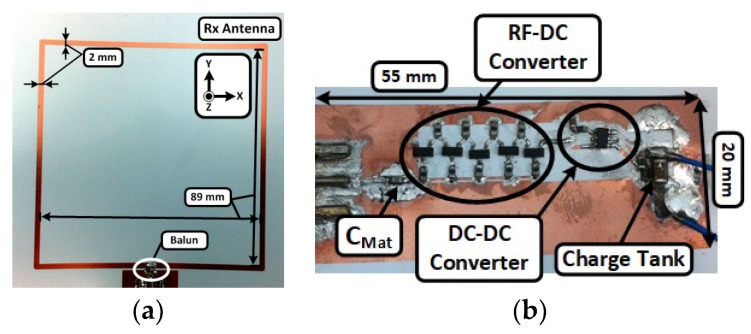
Fabricated RF energy harvesting system prototype utilizing the hybrid printed electronic technology: (**a**) square loop antenna and (**b**) RF-DC converter with a boost DC-DC converter.

**Figure 7:**
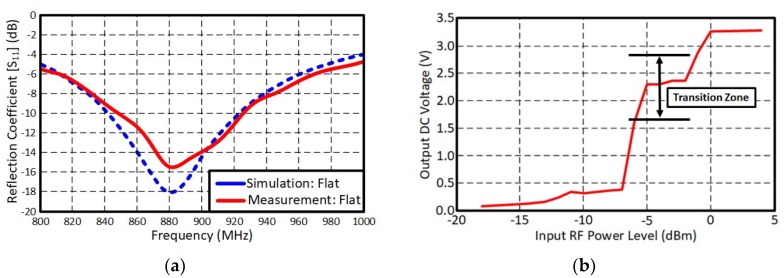
Measurement results. (**a**) The loop antenna and (**b**) RF-DC converter.

An optimally controlled thermal profile was used to attach components on the printed circuit layout. The thermal oven was preheated for 90 s at 130 °C to gradually increase the device temperature and activate the paste flux. Next, the temperature was increased to a maximum temperature of 155 °C for 55 s to allow the paste to flow. Afterwards, the device was slowly cooled down to 60 °C. This controlled heating profile allowed the paste to form a tight bond between the copper layer and the components without damaging the substrate. The fabricated RF energy harvester is shown in Figure 6 and all components have been successfully soldered on the printed thin copper film. The soldered component list is also shown in Table 1. Capacitance value for the charge tank (C_cp_) was 15 pF and Schottky diodes were used for the voltage rectification. A commercially available DC-DC converter was chosen for the voltage booster. Figure 7 shows the measured results of each component for the hybrid printed RF energy harvester system. The measured −10 dB bandwidth of the antenna covered the UHF RFID band at 880 ~ 915 MHz, and the RF energy harvester generated open circuit voltage of 3.3 V when the input RF power was above 0 dBm. When the input power level was from −7 dBm to −3 dBm, the RF-to-DC converter was in a ‘transition zone’. An output open circuit voltage swung from 1.6 V to 2.9 V at these input power levels (average voltage is shown in Figure 7b). Since the input DC voltage was lower than the turn-on voltage, the DC-DC boost converter at low input power levels could not generate stable output voltage. The output open-circuit voltage was also able to swing from 3.0 V to 3.8 V because of a floating drain when the output of the DC-DC boost converter was open (infinite output load impedance). The output voltage was stabilized when the converter was terminated by a load resister.

A 4.72 kΩ resistor was connected to the designed far-field RF energy harvester to validate the proposed autonomous battery-less self-sustainable wireless sensor system since the load resistor drained current of 190.7 μA at 900 mV (delivered DC power to the load is about 171.6 μW). The delivered power was high enough to drive an ultra-low power microcontroller unit (MCU) continuously because the ultra-low power MCU drains about 70 μA from a 900 mV voltage source at 1 MHz clock speed (MCU consumes about 63 μW) [17]. The measured voltage at the resistive load of 4.72 kΩ was about 900–950 mV when the received power by the loop antenna was higher than 0 dBm. The corresponding ambient power density was about 6.5 μW/cm^2^. It was demonstrated that the proposed RF energy transfer/harvesting system was able to power-up a low-power MCU continuously.

### 2.3. Direct Silver Nano-Particle Printing and Indirect Cssu Film Deposition

**Figure 8:**
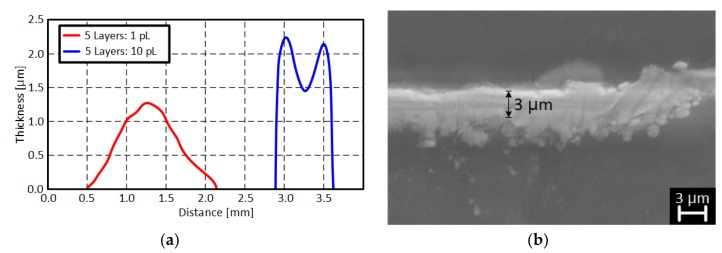
Cross-section of printed metal film: (**a**) Direct silver nano-particle printing method and (**b**) indirect thin copper film printing method.

**Figure 9:**
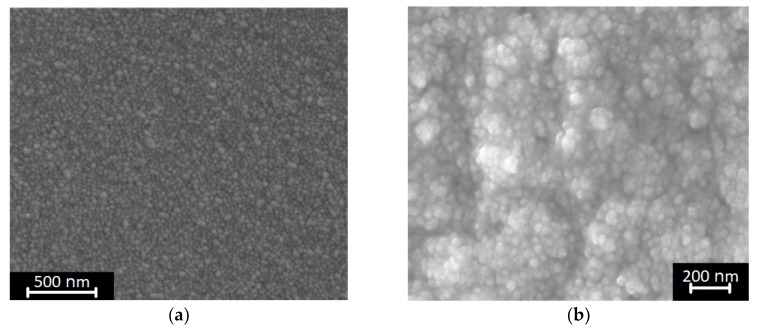
Surface SEM (Scanning Electron Microscope) images of (**a**) direct silver nano-particle printing method and (**b**) indirect thin copper film printing method.

**Figure 10:**
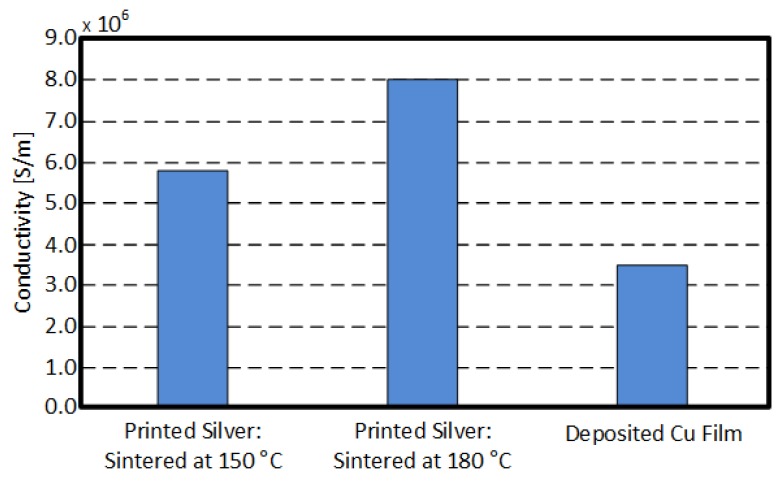
Conductivity values of the printed metal films.

In this section, the two metallization methods (direct and indirect printing) are discussed in detail. Both printing technologies deposit thin metallic layer which thickness is less than 5 μm as shown in Figure 8. Figure 8a shows the cross-section of the printed silver nano-particle ink for five times with 1 pL or 10 pL cartridge. The coffee ring effect was observed when a 10 pL droplet cartridge was used. The coffee ring effect occurs because of the different evaporation rate (drying rate) across the printed pattern or droplet. It can be suppressed by adding cellulose fiber, controlling the substrate temperature, or mixing low boiling point and high boiling point solvents [18,19,20]. A triangular cross-section was printed without the coffee ring effect for a 1 pL droplet cartridge because the amount of ink drop was small enough to dry at the same rate across the droplet. Figure 8b shows a SEM (Scanning Electron Microscope) image of the indirectly printed Cu film grown for 40 min in a CuSO_4_ bath. The thickness of the Cu film was 3 μm which was about 1 μm thicker than the directly printed silver nano-particle layer printed 5 times with a 10 pL cartridge. The measured surface roughness was about 14.4 nm for the directly printed silver nano-particle pattern and 250 nm for the indirect Cu film printing method. This was because the grain size of the grown Cu was much larger than the silver nano-particles as shown in the SEM images of the printed conductive films (Figure 9). The extracted electrical conductivity values are also shown in Figure 10. The directly printed silver nano-particle film sintered at 180 °C had a conductivity value of 8 × 10^6^ S/m but the grown Cu had about 3.5 × 10^6^ S/m. In summary, the inkjet-printed silver nano-particles had higher conductivity values and better surface roughness (smooth surface) than the indirectly printed Cu film. However, the indirect Cu printing technology was able to deposit a thicker metallic layer and soldering-compatible conductive film.

## 3. Conclusions

In this paper, a far-field energy harvesting system for self-sustainable wireless autonomous sensor applications was discussed. A wireless RF power supplier (active antennas) and far-field UHF electromagnetic energy harvester for battery-less autonomous sensors were critical components for a self-sustainable autonomous wireless sensor system. RF energy harvesting technology-based sensors converted electromagnetic power radiated from the RF power supplier to DC power to drive the microcontroller-based sensor system. Both devices were built using hybrid printed electronics technology. Two printing methods for metallization (direct nano-particle printing and indirect PdCl_2_ catalyst ink printing methods) were also presented and discussed in detail.

## Figures and Tables

**Table 1:** Component List.

Component	Value
Balun	100 Ω:50 Ω (balanced:unbalanced)
C_mat_	4.7 pF
C_cp_	15 pF
Diode	SMS7630 ^(1)^
DC-DC converter	TPS61073 ^(2)^
Charge tank	100 µF

^(1)^ Skyworks: http://skyworksinc.com/Products_Diodes.aspx; ^(2)^ Texas instruments: http://ti.com/product/tps61073.
